# The efficacy and safety of immunotherapy as first−line treatment for extensive-stage small cell lung cancer: evaluating based on reconstructed individual patient data

**DOI:** 10.3389/fonc.2024.1371313

**Published:** 2024-07-04

**Authors:** Shuang Zhang, Shuang Li, Ying Cheng

**Affiliations:** ^1^ Department of Thoracic Oncology, Jilin Cancer Hospital, Changchun, China; ^2^ Clinical Research Big Data Center, Jilin Cancer Hospital, Changchun, China

**Keywords:** immunotherapy, extensive-stage small cell lung cancer, first-line treatment, individual patient data, meta-analysis

## Abstract

**Objective:**

Selecting between programmed cell death ligand 1 (PD-L1) inhibitor or programmed cell death 1 (PD-1) inhibitor plus chemotherapy as first-line treatment for extensive-stage small cell lung cancer (ES-SCLC) patients urgently needs to be answered.

**Methods:**

Eligible phase 3 randomized clinical trials evaluating regimens based on PD-1/PD-L1 inhibitors as first-line treatment in ES-SCLC patients were systematically searched on the PubMed and Cochrane Library databases and major international conferences from 01/01/2018 to 18/09/2023. The individual patient data (IPD) were recuperated from the Kaplan–Meier curves of the overall survival (OS) and progression-free survival (PFS) of the included studies using the IPDfromKM method. The reconstructed data were pooled into unified arms, including the PD-L1 inhibitor plus chemotherapy group (PD-L1 group), PD-1 inhibitor plus chemotherapy group (PD-1 group), and PD-1 (L1) inhibitor and chemotherapy plus other (anlotinib group, tiragolumab group, and tremelimumab group). Subsequently, the PD-L1 group was indirectly compared with the other groups. A standard statistical analysis was conducted using the “survival” package for the time-to-event endpoint. The primary outcomes were the OS and PFS of the PD-L1 group and the PD-1 inhibitor group. The secondary outcomes included safety and the 12- and 24-month restricted mean survival time (RMST) of the PD-L1 group and PD-1 group.

**Results:**

A total of 9 studies including 11 immunotherapy cohorts were included. No significant difference in PFS (hazard ratio [HR]: 0.96, 95% confidence interval [CI]: 0.86–1.06), OS (HR: 0.94, 95% CI: 0.84–1.05), and 12-month and 24-month RMST for OS (P = 0.198 and P = 0.216, respectively) was observed between the PD-L1 group and the PD-1 group. In contrast, the anlotinib group showed significantly better OS (HR: 0.70, 95% CI: 0.55–0.89), PFS (HR: 0.69, 95% CI: 0.58–0.83), and RMST for OS compared to the PD-L1 group. The tiragolumab group showed similar efficacy to the PD-L1 group. However, the tremelimumab group exhibited inferior efficacy than the PD-L1 group. The incidence of ≥grade 3 treatment-emergent adverse events (TEAEs) was significantly higher in the PD-1 group compared to the PD-L1 group (85.4% vs. 69.6%, P <.001), whereas the incidence of irAEs was similar between the two groups.

**Conclusion:**

This reconstructed IPD analysis revealed that PD-1 inhibitors plus chemotherapy achieved similar efficacy to PD-L1 inhibitors plus chemotherapy as first-line treatment in ES-SCLC patients, whereas PD-L1 inhibitors plus chemotherapy had a better safety profile.

## Introduction

Small cell lung cancer (SCLC) is a highly aggressive subtype of lung cancer characterized by rapid proliferation and early dissemination, with a dismal prognosis (1, 2). At the initial diagnosis, approximately 70% of patients with extensive-stage small cell lung cancer (ES-SCLC) show involvement exceeding one hemithorax (3). The treatment options for ES-SCLC are very limited, and the first-line treatment of ES-SCLC has changed little over the past 40 years. Previous studies reported that the platinum combined with the etoposide (EP/EC) regimen as the standard first-line treatment achieved a median overall survival (OS) of only 10 months in ES-SCLC patients (4–6). However, as the focus of recent clinical research, immune checkpoint inhibitors (ICIs) represent therapeutic innovations in ES-SCLC.

Programmed cell death 1 (PD-1) and its ligand 1 (PD-L1) play an essential role as immune checkpoints. Blocking the PD-1/PD-L1 pathway can relieve the inhibition of T cells and exert antitumor effects. Although both PD-1 inhibitors and PD-L1 inhibitors target the PD-1/PD-L1 pathway, their mechanisms of action are different. PD-1 is mainly expressed on immune cells, with PD-L1 and PD-L2 being its two ligands (7, 8). PD-1 inhibitors block PD-1 on the surface of T cells to promote the immune system’s attack on tumor cells. PD-1 inhibitors also block the binding of PD-1 to PD-L2 (8). Compared with PD-L1, PD-L2 has a stronger affinity for PD-1 (8). In contrast, PD-L1 inhibitors lead to increased binding of PD-L2 to the repulsive guidance molecule B (RGMb), which may affect the homeostasis of the immune system and increase the risk of immune-related adverse events (9). PD-L1 is mainly expressed in tumor cells. In addition to blocking the binding of PD-L1 and PD-1, PD-L1 inhibitors also block the binding of PD-L1 on the surface of tumor cells and B7–1 on the surface of T cells, which promotes the activation of T cells (10). Furthermore, PD-L1 inhibitors can also inhibit the binding of B7–1 molecules on the surface of dendritic cells to their own PD-L1, thereby relieving the self-inhibition of dendritic cells and further enhancing the antitumor immune response (11). Moreover, PD-L1 inhibitors do not block the binding of PD-L1 to PD-L2 and reduce the occurrence of related adverse reactions.

Nonetheless, studies investigating the efficacy of PD-L1 inhibitors and PD-L1 inhibitors in SCLC patients reported inconsistent results. In 2018, the IMpower133 study (12) demonstrated for the first time that atezolizumab plus etoposide and carboplatin (EC) improved OS by approximately 2 months in ES-SCLC patients. Subsequently, the CASPIAN study (13) revealed that durvalumab plus chemotherapy had similar benefits for OS. Based on these two studies, atezolizumab or durvalumab plus chemotherapy were approved by the U.S. Food and Drug Administration (FDA) as a first-line treatment for ES-SCLC. Chemoimmunotherapy emerged as a new standard of care for the first-line treatment of patients with ES-SCLC. Recently, several confirmatory randomized controlled studies (RCTs) of first-line immunotherapy for ES-SCLC have been published. In general, the studies assessing the efficacy of PD-L1 inhibitors plus chemotherapy have achieved consistent results, demonstrating a prolonged OS of approximately 2 months compared with chemotherapy alone (12–15). However, the results of PD-1 inhibitors plus chemotherapy in first-line treatment for ES-SCLC were inconsistent. In the KEYNOTE-604 study, pembrolizumab plus chemotherapy only improved progression-free survival (PFS), whereas no statistical difference in OS was found (16); however, the addition of serplulimab to chemotherapy increased the OS by 4.5 months (17). Another PD-1 inhibitor, tislelizumab or toripalimab, combined with chemotherapy also showed an improvement in OS in ES-SCLC patients (18, 19).

Therefore, the differences in the efficacy and safety of PD-1 inhibitor or PD-L1 inhibitor plus chemotherapy as first-line treatment in ES-SCLC patients remain unelucidated. At present, the RCTs comparing the efficacy and safety of the two regimens are lacking. Meta-analyses were carried out to indirectly compare the efficacy of the two regimens. However, these two meta-analyses included only three phase 3 studies and overlooked recently published results (20, 21). In contrast, network meta-analysis can compare different regimes by integrating similar regimes (22–25). However, these network meta-analyses analyzed study-level data (22–25). As the gold standard for secondary analysis of data, the individual patient data (IPD) level of systematic reviews and meta-analyses is generally recognized (26, 27). The IPDfromKM method provides a stable and accurate method to obtain patient data from Kaplan–Meier curves (28–30). Therefore, the IPDfromKM method was employed to reconstruct IPD and indirectly compare the efficacy of first-line PD-1 inhibitors plus chemotherapy to PD-L1 inhibitors plus chemotherapy in ES-SCLC patients. With advances in immunotherapy in ES-SCLC, the treatment mode of addition other drug (“X” represent other drug) to PD-1(L1) inhibitor plus chemotherapy has emerged as an important research direction. Moreover, this study compared the efficacy of several “X” plus PD-1(L1) inhibitor and chemotherapy regimens to PD-L1 inhibitor plus chemotherapy regimens (31–33). Due to the unique pharmacokinetic characteristics of ICIs, the assumptions of proportional hazard (PH) proportionality are not met, so the hazard ratio (HR) of time-to-event endpoints (such as median OS) between two groups cannot be accurately calculated (34, 35). The restricted mean survival time (RMST) refers to the area under the survival curve under a certain time window (36–38), which can effectively represent the distribution of time events at any given time, ignoring the assumptions of PH (36, 39, 40). The RMST is an absolute measure of survival time and does not change with follow-up, which effectively explains the efficacy (35). Therefore, the RMST of OS was compared at the milestone time point of first-line immunotherapy across several regimens for ES-SCLC.

## Material and methods

This study adhered to the Preferred Reporting Items for Systematic Reviews and Meta-Analyses (PRISMA) Guidelines for IPD Systematic Reviews (41).

### Literature search

**Figure 1:**
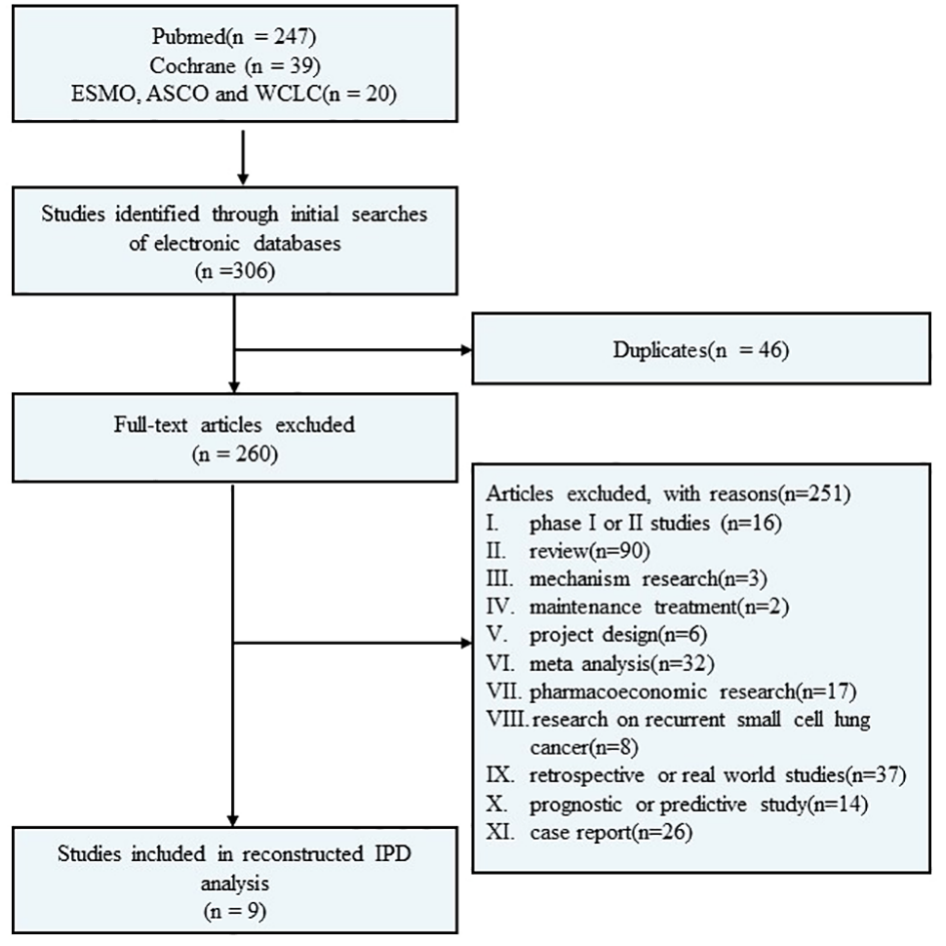
Flow diagram of our literature search and selection.

A systematic electronic search was conducted through the PubMed and Cochrane Library databases to identify randomized controlled phase III clinical trials investigating first-line immunotherapy for ES-SCLC. The search covered the period between 01/01/2018, and 18/09/2023. Additionally, abstracts from the European Society for Medical Oncology (ESMO), the American Society of Clinical Oncology (ASCO), and the World Conference on Lung Cancer (WCLC) since 01/01/2018 were also included in the search. The literature search was independently performed by the authors using specific search terms such as “small cell lung cancer OR SCLC,” “extensive disease,” “first-line treatment,” “PD-1/PD-L1,” “pembrolizumab,” “nivolumab,” “atezolizumab,” “durvalumab,” “avelumab,” “tremelimumab,” “tislelizumab,” “adebrelimab,” “serplulimab,” “benmelstobart,” “ipilimumab,” and “chemotherapy”. Furthermore, relevant references of eligible clinical trials were manually searched. Please refer to **Figure 1** for a detailed flow diagram.

### Inclusion and exclusion criteria

The inclusion criteria were as follows: 1. patients with ES-SCLC; 2 first-line treatment; 3. phase III RCTs; 4. treatment group acceptance PD-1/PD-L1 inhibitor-based combination treatment; 5. available Kaplan–Meier curves of OS, PFS, and safety data.

The exclusion criteria were as follows: 1. phase I, II studies; 2. observational or retrospective studies; 3. real-world studies; 4. studies of immune maintenance therapy after completion of standard chemotherapy; 5. non-randomized studies.

### Data extraction

Two researchers (Li S and Zhang S) extracted data independently, and the results were cross-checked; disparities were settled after evaluation by a third researcher. The following information was extracted from the included literature: name of study, phase of study, experimental arms regimen, number of patients, number of immunotherapy group patients, number of immunotherapy group PFS events, number of immunotherapy group OS events, median PFS of immunotherapy, median OS of immunotherapy, median follow-up time for OS, and adverse effects (AEs).

### Statistical analysis

Initially, WebPlotDigitizer (version 4.5 online; available at https://apps.automeris.io/wpd/) was employed to digitize the PFS and OS curves for 11 arms from the eight included studies, enabling extraction of step function values and timings. The graphical reconstructive algorithm developed by Liu et al. (28), which was based on the IPDfromKM method, was employed to reconstruct IPD from the survival curves for OS and PFS.

The data from studies targeting the same biological pathways, as determined by group consensus, were pooled into a unified arm for analysis. The 11 arms were further divided into five groups, including the PD-L1 group (represents pooled data of patients receiving PD-L1 inhibitors combined with chemotherapy as first-line treatment at the IPD level), the PD-1 group (represents pooled data of patients receiving PD-1 inhibitor plus chemotherapy at the IPD level), the anlotinib group (multitargeted tyrosine kinase inhibitor-anlotinib plus chemoimmunotherapy), the tiragolumab group (T-cell immunoreceptor with Ig and ITIM domain, TIGIT inhibitor-tiragolumab plus chemoimmunotherapy), and the tremelimumab group (cytotoxic T lymphocyte-associated antigen-4, CTLA-4 inhibitor-tremelimumab plus chemoimmunotherapy). In this study, the PD-L1 group was selected as the control group. The primary outcomes included the OS and PFS of the PD-L1 group and the PD-1 group. The secondary outcomes included the AE frequency (safety) and 12- and 24-month restricted mean survival time (RMST) of the PD-L1 group and the PD-1 group. Furthermore, the efficacy of the anlotinib group, the tiragolumab group, the tremelimumab group, and the PD-L1 group were evaluated as part of the exploratory analysis. A standard statistical analysis was performed using the “survival” package for the time-to-event endpoint. The HR with a 95% CI and medians of PFS and OS, along with their respective 95% CIs, were utilized as parameters in these analyses. In addition, the 12-month and 24-month RMST for OS were calculated for each group, and each group was compared to the PD-L1 group. Descriptive statistical analysis was used to calculate the incidence of AEs in each study, and the chi-square test was used to determine the difference in the incidence of AEs between the PD-L1 group and the PD-1 group.

All statistical analyses were performed using R-4.3.1 (http://www.r-project.org), and a level of *P* < 0.05 was considered statistically significant.

## Results

### Included clinical trials

A total of 306 studies were identified in the systematic screening. Finally, nine RCTs (12–19, 31–33) fulfilled the predetermined inclusion criteria and were included (**Figure 1**), comprising 11 immunotherapy cohorts and 2,677 patients, with 2,052 events of PFS and 1,666 events of OS. The detailed characteristics of the included studies are displayed in **Table 1**.

**Table 1:** Characteristics of included studies.

Study	Immunotherapy arm	Control arm	Total patients	No. of immunotherapy group patients	Immunotherapy group PFS events	Immunotherapy group OS events	Median PFS of immunotherapy	Median OS of immunotherapy	Median follow-up time for OS
PD-L1 group									
IMpower133 (12, 14)	Atezolizumab + EC	Placebo + EC	403	201	171	138	5.2 (4.4–5.6)	12.3 (10.8–15.8)	22.9 m
CASPIAN (31)	Durvalumab + EP/EC	EP/EC	805	268	234	210	5.1 (4.7–6.2)	12.9 (11.3–14.7)	25.1 m (IQR 22.3–27.9)
CAPSTONE-1 (15)	Adebrelimab plus EC	EC	462	230	175	151	5.8 (5.6–6.9)	15.3 (13.2–17.5)	13.5 m (IQR 8.9–20.1)
SKYSCRAPER-02 (32)	Placebo + atezolizumab plus EC	–	397	201	170	105	5.6 (5.4–5.9)	13.6 (12.3–15.2)	14.3 m
PD-1 group									
KEYNOTE-604 (16)	Pembrolizumab + EP/EC	Placebo + EP/EC	453	228	188	169	4.5 (4.3–5.4)	10.8 (9.2–12.9)	21.6 m (range, 16.1–30.6)
RATIONALE-312 (18)	Tislelizumab plus EP/EC	Placebo + EP/EC	457	227	175	164	4.8 (4.3–5.5)	15.5 (13.5–17.1)	14.2 m
ASTRUM-005 (17)	Serplulimab plus EC	Placebo + EC	585	389	223	146	5.7 (5.5–6.9)	15.4 (13.3–NE)	12.3 m (range, 0.2–24.8)
EXTENTORCH (18)	Toripalimab + EP	Placebo + EP	442	223	171	174	5.8 (5.6–6.8)	14.6 (12.9–16.6)	13.7 m
Tremelimumab group									
CASPIAN (31)	Durvalumab + tremelimumab + EP/EC	EP/EC	805	268	229	207	4.9 (4.7–5.9)	10.4 (9.6–12.0)	25.1 m (IQR 22.3–27.9)
Tiragolumab group									
SKYSCRAPER-02 (32)	Tiragolumab, atezolizumab plus EC	Placebo + atezolizumab plus EC	397	196	170	107	5.4 (4.7–5.5)	13.6 (10.8–14.9)	14.3 m
Anlotinib group									
ETER701 (33)	Benmelstobart with anlotinib plus EC	Placebo + placebo + EC	738	246	146	95	6.93 (6.18–8.25)	19.32 (14.23–NE)	14.0 m (range, 12.8–15.5)

Based on the characteristics of the investigational drug, the 11 cohorts were divided into five groups. The PD-L1 group comprised the atezolizumab plus EC arm from IMpower133, adebrelimab plus EC arm from CAPSTONE-1, durvalumab plus EC arm from CASPIAN, and atezolizumab and placebo plus EC arm from SKYSCRAPER02. The PD-1 group consisted of the serplulimab plus EC arm of ASTRUM-005, tislelizumab plus EP/EC arm of RATIONALE 312, pembrolizumab plus EP/EC arm of KEYNOTE-604, and toripalimab plus EP arm of EXTENTORCH. The durvalumab, tremelimumab plus EP/EC arm of CASPIAN constituted the tremelimumab group; the tiragolumab, atezolizumab plus EC arm of SKYSCRAPER02 constituted the tiragolumab group; and the benmelstobart, anlotinib plus EC arm of ETER701 constituted the anlotinib group.

The baseline characteristics of the PD-L1 group and the PD-1 group are reported in **Table 2**, showing a good numerical balance. The included patients were mostly aged<65 years, male, had an Eastern Cooperative Oncology Group (ECOG) score of 0 to 1, and were smokers. The proportion of patients with brain metastases was 10.1% in the PD-L1 group and 8.2% in the PD-1 group. The proportion of patients with liver metastases was 37.3% and 29.8% in these groups, respectively.

**Table 2:** Clinical characteristics of PD-L1 group and PD-1 group in the analysis.

Clinical characteristic	PD-L1 group	PD-1 group
Age		
<65 years	549 (58%)	632 (559.2%)
≥65 years	397 (42%)	435 (40.8%)
Sex		
Male	667 (70.5%)	838 (78.5%)
Female	279 (29.5%)	229 (21.5%)
ECOG score		
0	287 (30.3%)	208 (19.5%)
1	659 (69.7%)	859 (80.5%)
Smoking status		
Never	91 (9.6%)	190 (17.8%)
Current	270 (28.5%)	431 (40.4%)
Former	585 (61.8%)	446 (41.8%)
Brain metastasis	96 (10.1%)	87 (8.2%)
Liver metastasis	352 (37.3%)	318 (29.8%)

### Reconstructed survival curves and statistical comparisons

Considering that the PD-L1 inhibitor combined with chemotherapy is currently the internationally recognized standard first-line treatment of ES-SCLC, the PD-L1 group was used as the control group and was compared with other treatment groups.

### PD-1 group vs. PD-L1 group

#### OS

**Figure 2:**
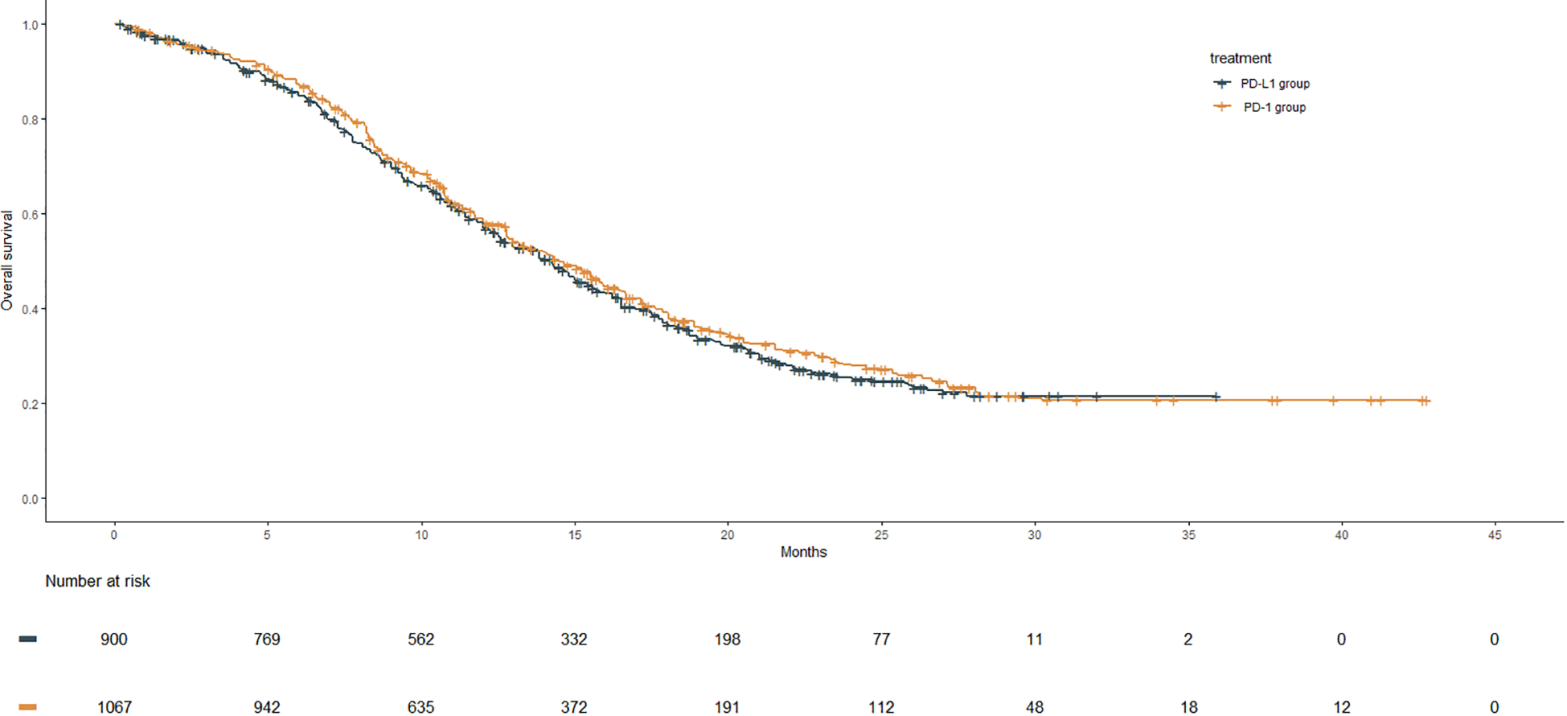
OS Kaplan–Meier curves from the reconstruction of IPD for PD-1 group compared with the PD-L1 group. The median OS of the PD-1 group and the PD-L1gourp were 14.5 m (95% CI: 13.4–15.5) and 14.3 m (95% CI: 13.0–15.0), respectively (HR: 0.94, 95% CI: 0.84–1.05, P = .280).

After reconstruction of IPD from the included trials, the Kaplan–Meier curves of OS for the PD-1 group were compared with those of the PD-L1 group, as shown in **Figure 2**. The PD-L1 group included 900 patients with a median OS of 14.3 months (95% CI: 13.0–15.0). The PD-1 group consisted of 1,067 patients, with a median OS of 14.5 months (95% CI: 13.4–15.5). However, no significant improvement in OS was observed in the PD-1 group compared with the PD-L1 group (HR: 0.94, 95% CI: 0.84–1.05, *P* = 0.280).

#### PFS

**Figure 3:**
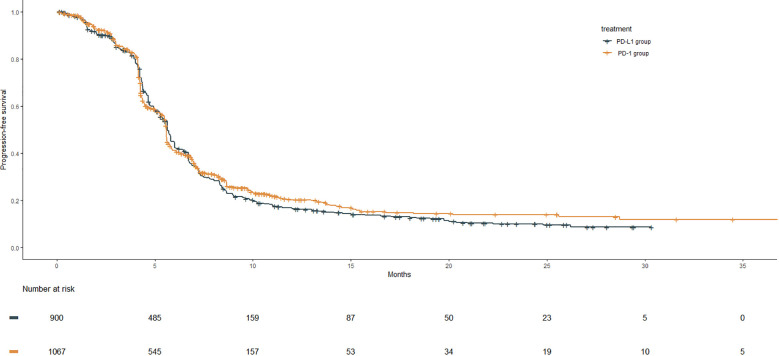
PFS Kaplan–Meier curves from the reconstruction of IPD for PD-1 group compared with the PD-L1 group. The median PFS of the PD-1group and the PD-L1gourp were 5.6 m (95% CI: 5.6–5.6) and 5.6 m (95% CI: 5.6–5.8), respectively (HR: 0.96, 95% CI: 0.86–1.06, P = .386).

The Kaplan–Meier curves of PFS for the PD-1 group were compared with those of the PD-L1 group, as shown in **Figure 3**. The median PFS of the PD-L1 group was 5.6 months (95% CI: 5.6–5.8), whereas that of the PD-1 group was 5.6 months (95% CI: 5.6–5.6), showing no significant benefit in the PD-1 group compared with the PD-L1 group (HR: 0.96, 95% CI: 0.86–1.06, *P* = 0.386).

#### RMST

Due to the significant crossing of OS and PFS survival curves between the two groups based on reconstructed IPD, the 12-month and 24-month RMST of OS were calculated in order to comprehensively evaluate the survival of the PD-1 group and the PD-L1 group (**Supplementary Table 1**). However, consistent with the trend of the median OS, the 12-month RMST (10.0 m vs. 9.9 m; difference: 0.19 m, 95% CI: −0.10 m to 0.47 m, P = 0.198) and the 24-month RMST (14.9 m vs. 14.5 m; difference: 0.44 m, 95% CI: −0.26 to 1.14 m, P = 0.216) of OS in the PD-1 group exhibited no statistically significant difference compared with that of the PD-L1 group.

### Safety

The incidence of treatment-emergent adverse events (TEAEs) and immune-related adverse events (irAEs) were compared between the PD-L1 group and the PD-1 group (**Supplementary Table 2** and **Supplementary Figure 1**). The results showed that the incidence of any grade TEAEs was slightly higher in the PD-L1 group than in the PD-1 group (99.4% vs. 98.2%, *P* = 0.035). The incidence of TEAEs was further analyzed by grade, demonstrating that the incidence of grade 1–2 TEAEs was also higher in the PD-L1 group than in the PD-1 group (29.7% vs. 12.8%, *P* < 0.001). In contrast, the incidence of grade ≥3 TEAEs (85.4% vs. 69.6%, *P* < 0.001) and TEAEs leading to death (6.6% vs. 4.0%, *P* = 0.016) was significantly higher in the PD-1 group compared with the PD-L1 group. No significant difference was observed in any serious TEAEs between the two groups (*P* = 0.152). The incidence of irAEs was 33.8% in the PD-L1 group and 33.0% in the PD-1 group, showing no statistically significant difference (*P* = 0.751). Furthermore, the incidence of grade 1–2 irAEs and grade ≥3 irAEs was reported as 25.8% and 6.3% in the PD-L1 group and 22.5% and 9.0% in the PD-1 group, respectively, with no statistical difference (*P* = 0.150, *P* = 0.062).

### “X” plus PD-1(L1) inhibitor and chemotherapy groups vs. the PD-L1 group

#### OS

The tremelimumab group, consisting of 268 patients, exhibited a median OS of 10.4 months (95% CI: 9.6–12.0). In comparison with the PD-L1 group, the tremelimumab group demonstrated inferior OS (HR: 1.22, 95% CI: 1.04–1.43, *P* = 0.014). The tiragolumab group consisted of 196 patients and had a median OS of 13.6 months (95% CI: 10.8–14.9). No significant improvement in OS was observed in the tiragolumab group compared with the PD-L1 group (HR: 1.05, 95% CI: 0.85–1.30, *P* = 0.649). The anlotinib group comprised 246 patients and demonstrated a median OS of 19.3 months (95% CI: 14.2–NE). The anlotinib group exhibited significantly superior OS compared with the PD-L1 group, with an HR of 0.70 (95% CI: 0.55–0.89, *P* = 0.003) (**Supplementary Figure 2**).

#### PFS

The median PFS of the tremelimumab group was 4.9 months (95% CI: 4.7–5.9), which showed no statistically significant difference between that of the PD-L1 group (HR: 1.05, 95% CI: 0.90–1.21, *P* = 0.561). The median PFS was 5.4 months (95% CI: 4.7–5.5) in the tiragolumab group, demonstrating no significant PFS benefit compared with the PD-L1 group (HR: 1.14, 95% CI: 0.96–1.35, *P* = 0.132). The median PFS of the anlotinib group was 6.9 months (95% CI: 6.2–8.3). The anlotinib group achieved a significantly better PFS than the PD-L1 group (HR: 0.69, 95% CI: 0.58–0.83, *P* < 0.001) (**Supplementary Figure 3**).

#### RMST

Consistent with the benefit trend of OS, the anlotinib group showed a significantly superior 12-month RMST (10.4 m vs. 9.9 m; difference: 0.54 m, 95% CI: 0.12 to 0.96 m, P = 0.012) and 24-month RMST (16.6 m vs. 14.5 m; difference: 2.12 m, 95% CI: 0.75 m to 3.48 m, P = 0.002) compared with the PD-L1 group. In contrast, the tremelimumab group exhibited a significantly poorer 12-month RMST (9.0 m vs. 9.9 m; difference: −0.86 m, 95% CI: −1.35 to −0.37 m, *P* = 0.001) and 24-month RMST (12.8 m vs. 14.5 m; difference: −1.69 m, 95% CI: −2.78 to −0.60 m, *P* = 0.002) compared with the PD-L1 group. However, the 12-month (*P* = 0.789) and 24-month RMST (*P* = 0.571) for OS in the tiragolumab group showed no statistically significant difference compared with the PD-L1 group (**Supplementary Table 3**).

## Discussion

For the first time, this study compared the efficacy and safety of PD-1 inhibitors plus chemotherapy to that of PD-L1 inhibitors plus chemotherapy at the IPD level by integrating data from nine phase III studies. In this IPD meta-analysis, no statistically significant differences in PFS and OS were observed between PD-1 inhibitors or PD-L1 inhibitors plus chemotherapy as first-line treatment of ES-SCLC. The 12-month and 24-month RMST for OS also revealed similar findings. From a safety perspective, the incidence of any grade TEAEs was slightly higher in the PD-L1 group than in the PD-1 group, which primarily consisted of a higher incidence of grade 1–2 TEAEs in the PD-L1 group. However, the PD-1 group had a higher incidence of ≥ grade 3 TEAEs and TEAEs leading to death compared with the PD-L1 group. Nevertheless, the incidence of serious TEAEs and irAEs was comparable between both groups. Synthetic data from the PD-L1 inhibitors plus chemotherapy group were used as a control, and the efficacy of several “X” plus chemoimmunotherapy regimens was evaluated. The results revealed that benmelstobart with anlotinib plus EC exhibited significantly improved OS and PFS compared with the PD-L1 group. The OS and PFS of the tiragolumab group were similar to the PD-L1 group, whereas the efficacy of the durvalumab and tremelimumab plus EC was inferior to the PD-L1 group. The results also suggested not all of the “X” plus PD-1(L1) inhibitor and chemotherapy has the effect of icing on the cake. In the absence of head-to-head RCTs comparing PD-L1 inhibitors with PD-L1 inhibitors plus chemotherapy in the first-line treatment of ES-SCLC, this study provides the available high-level evidence for clinical decision.

Several unique features distinguish our study from previous meta-analyses in immunotherapy for ES-SCLC. To our knowledge, this is the first meta-analysis investigating the efficacy of ICI-based regimens as first-line treatment for ES-SCLC employing reconstructed IPD. The efficacy of different ICI-based regimens as first-line treatment for ES-SCLC was granularly assessed. Although PD-1 inhibitor or PD-L1 inhibitor plus chemotherapy as the first-line treatment for ES-SCLC was being investigated, the results of PD-L1 inhibitors and PD-1 inhibitors were differences in ES-SCLC. A PD-1 inhibitor-serplulimab and several PD-L1 inhibitors (atezolizumab, durvalumab, and adebrelimab) have been approved as first-line treatment for ES-SCLC in China. This raises the need to compare the efficacy of PD-1 inhibitors to PD-L1 inhibitors to improve the first-line treatment for ES-SCLC. An ongoing phase III head-to-head study compares the PD-L1 inhibitor serplulimab to the PD-L1 inhibitor atezolizumab plus chemotherapy as first-line treatment for ES-SCLC (NCT05468489). In the absence of direct comparative results, indirect comparative results based on this IPD meta-analysis play a critical role in clinical decision-making. Furthermore, RMST was analyzed to evaluate the OS of different ICI-based regimens to avoid bias in the Kaplan–Meier curves against the PH assumption. Our previous study revealed that the 12-month and 24-month RMST for OS were strongly correlated with the median OS in first-line immunotherapy for ES-SCLC (42). A growing number of studies on immunotherapy also analyzed RMST as an exploratory endpoint (15, 16). In our study, no absolute difference in 12-month RMST for OS was found between the PD-1 inhibitor regimen and the PD-L1 regimen. However, the 24-month RMST for OS of the PD-1 inhibitor regimen tended to be longer than that of the PD-L1 inhibitor regimen, although the difference was not statistically significant. These results are consistent with our analysis of the HR of OS on the synthetic data. Finally, our analysis included studies of innovative immunotherapy regimens, particularly the ETER701 study, which found a significant PFS and OS benefit with benmelstobart and anlotinib plus EC; however, the control group in the ETER701 study only consisted of chemotherapy. Therefore, the results of the ETER701 study cannot conclude whether benmelstobart and anlotinib plus EC were superior to PD-L1 inhibitors plus chemotherapy. Still, our reconstructed IPD analysis indicated that the benmelstobart and anlotinib plus EC group was significantly more effective than the PD-L1 group. Additionally, the reduce risks of disease progression (31%) and death (30%) were similar in the anlotinib group to the PD-L1 group. Notably, head-to-head studies comparing innovative immunotherapeutic regimens with standard immunotherapeutic regimens are also in progress (NCT05844150; NCT05224141), which will provide more direct evidence in the future. At present, the indirect comparison through IPD analysis provides evidence to make clinical decisions. The new standard-of-care immunochemotherapy may not have been used as a control in some studies due to research funding or immunotherapy not being approved at the start of the study.

### Limitations

Nevertheless, the limitations of the current study should be acknowledged. First, although the PFS and OS of the PD-L1 inhibitors plus chemotherapy and the PD-1 inhibitors plus chemotherapy groups were accurately evaluated by reconstructing IPD, the algorithm was unable to capture variables other than survival data. As a result, important study-level covariates that may have impacted OS and PFS could not be fully evaluated. Moreover, the Kaplan–Meier curves of subgroups in most studies could not be obtained, which hindered further subgroup analysis. Second, pooled analyses were only performed for the incidence of TEAEs and irAEs. Due to the heterogeneity of published safety data, the safety assessment of different treatment regimens remains incomplete. Thirdly, the published results of “X” plus PD-1(L1) inhibitor and chemotherapy in ES-SCLC remain limited. Currently, only one phase 3 study has been published for each innovative regimens. In addition, studies comparing the safety between “X” plus chemoimmunotherapy and chemoimmunotherapy are lacking. Our results regarding the efficacy of the “X” plus chemoimmunotherapy regimen compared with immunotherapy need to be interpreted with caution. Different conclusions may be reached as more research results are published in the future. Furthermore, our study showed that the addition of CTLA-4 inhibitors to chemoimmunotherapy was not superior to chemoimmunotherapy. The results also highlight the need for more mechanistic studies to explore rational immunotherapy strategies, rather than simply adding existing accessible drugs. Finally, cost-effectiveness analysis is also an indispensable factor for evaluating the clinical significance of a regimen and making clinical decisions. However, the present study conducted no such analysis.

## Conclusion

In conclusion, the efficacy of PD-L1 inhibitors in combination with chemotherapy was equivalent to that of PD-1 inhibitors in combination with chemotherapy as the first-line treatment for ES-SCLC, whereas PD-L1 inhibitors plus chemotherapy had better safety. In addition, anti-angiogenesis agents in combination with chemoimmunotherapy may be a more effective first-line treatment for patients with ES-SCLC, but more data are required.

## Data availability statement

The original contributions presented in the study are included in the article/**Supplementary Material**. Further inquiries can be directed to the corresponding author.

## Author contributions

SZ: Writing – original draft, Writing – review & editing. SL: Writing – original draft. YC: Writing – original draft, Writing – review & editing.
